# Evaluation of SMOS, SMAP, AMSR2 and FY-3C soil moisture products over China

**DOI:** 10.1371/journal.pone.0266091

**Published:** 2022-04-07

**Authors:** Jiazhi Fan, Man Luo, Qinzhe Han, Fulai Liu, Wanhua Huang, Shiqi Tan

**Affiliations:** 1 China Meteorological Administration Training Centre Hunan Branch, Hunan Meteorological Bureau, Changsha, China; 2 Key Laboratory of Hunan Province for Meteorological Disaster Prevention and Mitigation, Hunan Meteorological Bureau, Changsha, China; 3 International Center for Ecology, Meteorology and Environment, Jiangsu Key Laboratory of Agricultural Meteorology, Nanjing University of Information Science and Technology, Nanjing, China; 4 Hunan Research Institute of Meteorological Sciences, Hunan Meteorological Bureau, Changsha, China; 5 Hunan Meteorological Service Center, Hunan Meteorological Bureau, Changsha, China; National Research Council, ITALY

## Abstract

Microwave remote sensing can provide long-term near-surface soil moisture data on regional and global scales. Conducting standardized authenticity tests is critical to the effective use of observed data products in models, data assimilation, and various terminal scenarios. Global Land Data Assimilation System (GLDAS) soil moisture data were used as a reference for comparative analysis, and triple collocation analysis was used to validate data from four mainstream passive microwave remote sensing soil moisture products: Soil Moisture and Ocean Salinity (SMOS), Soil Moisture Active and Passive (SMAP), Global Change Observation Mission–Water using the Advanced Microwave Scanning Radiometer 2 (AMSR2) instrument, and Fengyun-3C (FY-3C). The effects of topography, land cover, and meteorological factors on the accuracy of soil moisture observation data were determined. The results show that SMAP had the best overall performance and AMSR2 the worst. Passive microwave detection technology can accurately capture soil moisture data in areas at high altitude with uniform terrain, particularly if the underlying surface is soil, and in areas with low average temperatures and little precipitation, such as the Qinghai–Tibet Plateau. FY-3C performed in the middle of the group and was relatively optimal in northeast China but showed poor data integrity. Variation in accuracy between products, together with other factors identified in the study, provides a baseline reference for the improvement of the retrieval algorithm, and the research results provide a quantitative basis for developing better use of passive microwave soil moisture products.

## 1. Introduction

Water is an important resource on earth and a key component of the continuous soil–vegetation–atmosphere hydrologic cycle [1]. Soil moisture (SM) is a critical variable in the carbon and water cycles that serves as an important index of surface water and the energy budget [2]. SM is the main source of water for plants [3]. Accurate SM data is necessary for science-based agricultural production [4] as it increases the value of research into the water cycle and energy fluxes, increases the accuracy of climate and weather forecasting [5], and makes disaster warnings more reliable [6].

The principal methods of SM data acquisition currently used are automated instrumental observation and microwave remote sensing. Instrumental observation data are accurate, but the spatial resolution of the data often does not meet current demands for data. Microwave remote sensing of SM provides a wide range of surface information [7, 8] and it is very efficient; it provides data over a wide area [9, 10] and is unaffected by weather conditions, in addition to providing long-term SM data [11, 12]. These benefits have ensured that it has rapidly become a primary SM data source.

Widely used satellite-borne passive microwave remote sensing SM observation products include the following: data observed by the microwave imaging radiometer with aperture synthesis carried on the European Space Agency Soil Moisture and Ocean Salinity (SMOS) mission; data observed by the microwave radiometer carried on the U.S. NASA Soil Moisture Active Passive (SMAP) mission; data observed by the advanced microwave scanning radiometer 2 (AMSR2) carried on the joint America–Japan Global Change Observation Mission–Water; and data observed by the microwave radiation imager (MWRI) carried on the Chinese Fengyun-3C mission. These four products provide important data to support hydrometeorology research and disaster prevention and mitigation [13, 14], but there is no consensus on the applicability of these products in research on a regional scale, and this uncertainty reduces the usefulness of the products.

A comprehensive assessment and evaluation of the performance of remote sensing SM products can lead to improved quality and increase their value in research into climate, hydrology and natural disasters. Different reference data (ground observation network data, data for core validation sites, comparisons between satellite missions, and model simulations, among others) have been used in numerous validation and evaluation studies [15–18]. The reference data used in relevant studies offer both advantages and disadvantages: ground observation data is real and accurate, but the spatial scale based on a single station is quite different from the elliptical spatial grids of remote sensing products. The uneven distribution of ground observation stations across different regions leads to large differences in the amounts of available data for different environmental conditions, which affects the utility of the regional representation of assessment results. Assimilated data offers uniform grid distributions and consistent time series, so the use of assimilated data as a baseline can offer a more accurate comparison of remote sensing products from satellite observations.

Methods of validation and evaluation of soil moisture products can be divided into direct evaluation based on multiple error coefficients and triple collocation analysis based on decomposition error components. The results of the direct evaluation are intuitive and easy to understand but their accuracy depends on the quality of the reference data. Triple collocation analysis (TCA) is a method of estimating the random error variation of three collocated datasets of the same geophysical variable [19]. It does not require an available high-quality reference dataset and has therefore developed into an important evaluation tool in earth observation. All metrics from TCA-based validation theoretically lie between the soil moisture product under evaluation and the unknown truth [20], which allows for a more equitable evaluation of the accuracy and error characteristics of different soil moisture products [21, 22].

Studies of verification, the application of new theories, and the development of improved methods of parameterization have all improved remote sensing retrieval algorithms used to quantify SM [23]. However, many comprehensive remote sensing SM product evaluation studies lacked any assessment of data accuracy for Asia, particularly China [17]. The geographic heterogeneity of China makes it a suitable region for a comprehensive comparison and assessment of SM products under different climatic and environmental conditions. The objective of this study was to develop a method of comprehensive evaluation that used assimilated datasets as baseline reference data to determine the authenticity of mainstream satellite-borne passive microwave remote sensing soil moisture products for China. We identified the effects of topography, land cover classification and meteorological factors on data authenticity. The errors in each product were decomposed and analyzed using TCA. The results obtained provide a reference for future improvement of remote sensing retrieval algorithms. This study also provides a scientific basis for the development and application of remote sensing SM products and provides data to support science-based agricultural production. Government departments can benefit from our research through improved drought and flood monitoring at a local scale, which enables better decision making. This research also offers technical support for research in meteorology and hydrology.

## 2. Materials and methods

### 2.1 Study area

China covers a land area of 9.6 million km^2^, spanning 52 degrees of latitude from north to south and 63 degrees of longitude from east to west. China embraces tropical, subtropical and temperate zones, and humid, sub-humid, semi-arid and arid regions in its vast land area with a variety of regional climates. China’s terrain is high in the west and low in the east. The Qinghai–Tibet Plateau in the west of the country has an average elevation of more than 4000 meters above sea level. The terrain of the country is complex and diverse; mountains, plateaus and hills account for about two-thirds of the land area, and basins and plains account for about one-third.

### 2.2 Soil moisture products

The passive microwave soil moisture products used in this study were from four satellites, and the data were retrieved as daily data products from 2017-01-01 to 2019-12-31 (Table 1). SMOS was the world’s first satellite mission to measure soil moisture content on the planet surface and its soil moisture products are widely used [24]. Observation was by L-band (1.4 GHz) detection with a microwave imaging radiometer (MIRAS); resolution was 27–55 km, and induction depth was 3–5 cm. The SMOS-L3-SM product is gridded soil moisture data obtained after spatiotemporal recombination of L2 soil moisture data; resolution is 25 km and the Equal-Area Scalable Earth Grid 2.0 (EASE-Grid 2.0) is used. The data version is V3.0, the download website is www.catds.fr/, and the data is in netCDF format.

**Table 1:** Overview of the four satellite soil moisture datasets being compared.

	SMOS	SMAP	AMSR2	FY-3C
Satellite	SMOS	SMAP	GCOM-W	FY-3C
Sensor	MIRAS	radiometer	AMSR2	MWRI
Time period	Jun 2010–present	Mar 2015–present	Jul 2012–present	May 2014–present
Band frequency	1.4 GHz	1.4 GHz	10.7 GHz	10.7 GHz
Spatial sampling	25 km EASE-2	36 km EASE-2	25 km	25 km EASE
Sensor resolution	27–55 km	43 km	24–42 km	50–75 km
Spatial coverage	Global	Global	Global	Global
Asquisition time (local time)	ASC: 06:00	ASC: 18:00	ASC: 13:30	ASC: 22:00
	DES: 18:00	DES: 06:00	DES: 01:30	DES: 10:00
Product version	SMOS-L3 V3.0	SMAP-L3 V7	LPRM AMSR-2 L3 V001	FY-3C/MWRI V1.0.0
Unit	m^3^/m^3^	m^3^/m^3^	%	cm^3^/cm^3^

Note: All periods in the data are as of Jan 2020; DES is descending and ASC is ascending.

SMAP is the latest satellite that is dedicated to soil moisture detection, and its L-band soil moisture products are widely used in climate and environmental monitoring. The passive remote sensing L3 product has a resolution of 36 km, and the projection is EASE-Grid 2.0. The data version is V7, the download website is https://nsidc.org/, and the data is in HDF format.

AMSR-E/2 soil moisture retrieval products are widely used in various fields. The AMSR-2 L3 X-band (10.7 GHz) soil moisture product from the Land Parameter Retrieval Model (LPRM) can avoid radio frequency interference (RFI). It has a resolution of 25 km. The data version is V001, the download website is https://disc.gsfc.nasa.gov, and the data is in netCDF format.

FY-3C is the third mission of the FY-3 series of second-generation polar orbiting meteorological satellite missions. The FY-3 series was independently developed by China. FY-3C observation products are widely used in weather forecasting, climate change monitoring, and in applications that support agriculture, transportation, shipping and other fields. Soil moisture data were observed by dual polarized X-band (10.7 GHz) detectors. The National Satellite Meteorological Center of China provides FY-3C/MWRI daily soil moisture products with a resolution of 25 km. The projection is EASE-Grid. The data version is V1.0.0, the download site is http://satellite.nsmc.org.cn/, and the data is in HDF format.

The problem of scale mismatch in remote sensing product verification is always difficult to solve. In this study, we used the Global Land Data Assimilation System (GLDAS), a globally available reference soil moisture assimilation dataset, to reduce test errors caused by scale differences. GLDAS is the most widely used land surface data assimilation product, which is ingested by satellite- and ground-based observational data products and generated with advanced land surface modeling and data assimilation techniques [25]. GLDAS soil moisture products are used in hydrology and in water cycle and climate change research at large watershed and global scales. We used the 3-hourly GLDAS-2.1 Noah-3.6 product, downloaded from https://disc.gsfc.nasa.gov, with data in netCDF format. Soil moisture data for the 0–10 cm layer was extracted from the product to be used as the reference dataset. Spatial resolution was 0.25°×0.25°. Auxiliary data used in data preprocessing included GLDAS-2.1 soil temperature for the 0–10 cm layer and the snow depth dataset.

### 2.3 Other auxiliary data

Auxiliary environmental data for the study area were also collected for our comprehensive comparison, analysis and evaluation of the four SM products. The auxiliary topographic data included NASA’s Advanced Spaceborne Thermal Emission and Reflection Radiometer Global Digital Elevation Model Version 3 (GDEM) 30 m resolution elevation datasets (https://doi.org/10.5067/ASTER/AST14DEM.003). Auxiliary land cover classification data was obtained from the Multi-Period Land Use Land Cover Remote Sensing Monitoring Dataset (CNLUCC) for 2018 with a resolution of 1 km, downloaded from the Institute of Geographic Sciences and Natural Resources Research, Chinese Academy of Sciences (https://www.resdc.cn/data.aspx?DATAID=264). Auxiliary precipitation data was taken from the China Precipitation Daily 0.5°×0.5° Grid Data (V2.0) of the China Meteorological Administration (http://data.cma.cn/). The 0–10 cm soil temperature data in the GLDAS dataset was used as the surface temperature (T_s_) data.

### 2.4 Methods

#### 2.4.1 Data preprocessing

We attempted to process all data products uniformly to avoid systematic errors affecting the evaluation. The FY-3C product does not contain data quality control flags, so the data from all four satellites were not filtered due to data quality control parameters. However, the dielectric properties of water and ice are significantly different [26], and the capability of microwave sensing to detect soil moisture is limited for freezing water. Therefore, frozen season data were eliminated according to soil temperature values and snow depth values for the underlying surface during the transit time of satellite observation data collection [27].

Data were preprocessed in the following stages.

1. The data units of SM products for the period 2017–2019 were uniformly converted to volumetric water content (cm^3^/cm^3^). The SM parameters were re-projected into the GLDAS grid; nearest-neighbor sampling was used for SMOS and FY-3C [20], and inverse distance weighting was used for SMAP [28]. A satellite remote sensing observation datum has an associated instantaneous observation time; temporal nearest neighbor matching between remote sensing products and GLDAS data based on the transit time of each remote sensing satellite was used in the authenticity test. Unmatched data and data from footprints with <5 data pairings after matching were not considered in subsequent analysis. Data were screened with reference to GLDAS snow depth and soil temperature data for the observation periods of the subject datasets, and data for snow depth >0.1 m or soil temperature <0°C were removed. The performance of each of the four soil moisture products was evaluated for each matched dataset and the accuracy of the observation of soil moisture in China was determined for each product.

2. GDEM data and CNLUCC data were resampled to the GLDAS grid, and the means, ranges and standard deviations of elevation, modal number and information entropy of land cover classification in each grid were calculated.

Information entropy is a measure of system uncertainty. The probability distribution of a random variable *X* with *n* possible outcomes is *P*(*X* = *x*_*i*_) = *p*_*i*_ for *i* = 1,2,…,*n*. Information entropy *H*(*X*) is calculated by:

$$H(X)=-\overset{n}{\underset{i=1}{\sum }}p_{i}\log p_{i}$$ (1)

Precipitation data were also resampled to the GLDAS grid, and the means, standard deviations, maxima and medians of daily precipitation and the means, standard deviations, maxima, minima and ranges of *T*_*s*_ data in each grid were calculated.

The effects of topography, landcover type, and meteorological conditions on the accuracy of remote sensing SM products were determined.

3. The optimal product of the four remote sensing SM products and GLDAS data were temporally and spatially matched with the other three products for TCA. The matched dataset was at a daily time step on the GLDAS grid and error components for this dataset were calculated and analyzed.

#### 2.4.2 Performance index

1. Authenticity test

Correlation analysis was used to evaluate the performance of different remote sensing soil moisture products in China-wide observations. We compared the results of ascending orbit observations, descending orbit observations and regional observations and analyzed the effects of environmental factors on the accuracy of each product. Four statistical measures, widely used to test the authenticity of SM products, were used to evaluate the performance of each product: the Pearson correlation coefficient (*r*; Eq (2)) to indicate the accuracy of satellite-based observations to match variation in SM in GLDAS datasets; relative bias (Bias_r_; Eq (3)) to measure the extent to which the retrieval parameters of spaceborne sensors were dry or wet relative to GLDAS data; root mean square error (RMSE; Eq (4)) to indicate the deviation between remote sensing soil moisture data and GLDAS data; and unbiased root mean square error (ubRMSE; Eq (5)) to remove the effect of random error on RMSE and better measure absolute deviation. The four indicators were calculated by:

$$r=\frac{\mathrm{cov}(RSSM,ASSM)}{\sigma_{RSSM}\sigma_{ASSM}}$$ (2)

$${\mathrm{Bias}}_{r}=\frac{1}{m}\overset{m}{\underset{i=1}{\sum }}\frac{\left(RSSM_{i}-ASSM_{i}\right)}{ASSM_{i}}$$ (3)

$$RMSE=\sqrt{\frac{1}{m}\overset{m}{\underset{i=1}{\sum }}{\left(RSSM_{i}‐ASSM_{i}\right)}^{2}}$$ (4)

$$ubRMSE=\sqrt{RMSE^{2}-{\left(\frac{1}{m}\overset{m}{\underset{i=1}{\sum }}(RSSM_{i}-ASSM_{i})\right)}^{2}}$$ (5)

where RSSM is SM of each remote sensing product, ASSM is SM of the GLDAS product, *cov*() is covariance, and *σ* is standard deviation.

2. Triple collocation analysis

The following three parameters were used to indicate the results of TCA. Sensitivity (Eq (6)) indicates the sensitivity of the product to the real signal changes in soil moisture. Standard error (Stderr; Eq (8)) indicates standard errors for each of the measurement systems [29]; signal-to-noise ratio in decibels (Snr_db; Eq (10)) indicates the ratio of signal to noise in logarithmic form.

$${S\mathrm{ensiti}\mathrm{vity}}_{a}=\frac{\mathrm{cov}(a,b)\times \mathrm{cov}(a,c)}{\mathrm{cov}(b,c)}$$ (6)

$$\mathrm{Err}{\mathrm{var}}_{a}=\mathrm{cov}(a,a)-{\mathrm{Sensitivity}}_{a}$$ (7)

$$\mathrm{If}\;\mathrm{Err}{\mathrm{var}}_{a}\geq 0,\;\mathrm{then}\;{\mathrm{Stderr}}_{a}=\sqrt{{\mathrm{Err}\mathrm{var}}_{a}}$$ (8)

$$Snr_{a}=\frac{\mathrm{cov}(a,a)\times \mathrm{cov}(b,c)}{\mathrm{cov}(a,b)\times \mathrm{cov}(a,c)}-1$$ (9)

$${\mathrm{If}\;\mathrm{Snr}}_{a}\geq 0,\;\mathrm{then}\;\mathrm{Snr}\_db_{a}=-10\times \log(Snr_{a})$$ (10)

where *a*, *b* and *c* are three SM products matched in daily time scale and GLDAS grid step, and cov() is covariance.

Of the preceding parameters, greater *r* and Sensitivity and lesser Bias_r_, RMSE, ubRMSE and Stderr indicate better product performance. A value of zero for Snr_db indicates that signal variance is equal to noise variance; +3(+6) dB indicates that signal variance is twice (four times) noise variance; −3(−6) dB indicates that signal variance is half (one fourth) noise variance, and so forth [30].

## 3. Results

### 3.1 Comparative analysis of authenticity of remote sensing soil moisture data

The indicators *r*, Bias_r_, RMSE, and ubRMSE were computed between each remotely-sensed SM dataset (after data screening) and the GLDAS data (Table 2). All available observations within the 2017–2019 period were included. Thus the period used to compute the scores for each product was consistent across products.

**Table 2:** Indicator values for comparison of remote sensing data with GLDAS data.

		*r*	Data quantity (million)	Bias_r_ (%)	RMSE (cm^3^/cm^3^)	*ub*RMSE (cm^3^/cm^3^)
SMOS	ASC	0.5	2.94	−44.69	0.133	0.096
	DES	0.52	2.59	−45.25	0.137	0.096
	all	0.51	5.53	−44.95	0.135	0.096
SMAP	ASC	0.73	*1*.*18*	**−13.47**	0.09	0.085
	DES	**0.77**	1.85	−15.45	**0.078**	**0.071**
	all	**0.76**	*3*.*03*	**−14.68**	**0.083**	**0.077**
AMSR2	ASC	*0*.*38*	**9.35**	41.18	0.235	0.222
	DES	0.5	8.14	*60*.*8*	*0*.*276*	*0*.*235*
	all	*0*.*44*	**17.5**	*50*.*31*	*0*.*255*	*0*.*231*
FY-3C	ASC	0.51	4.28	−25.51	0.125	0.11
	DES	0.5	5.09	−27.39	0.123	0.106
	all	0.51	9.37	−26.53	0.124	0.108

Note: Data in bold are the optimal parameter values overall and for each orbit; data in italic are the least optimal; DES is descending and ASC is ascending.

Overall analysis shows that during the three years 2017–2019, SMAP data were significantly more accurate for China than data from the other three products, as indicated by the optimal values of *r*, Bias_r_ (in absolute value), RMSE and ubRMSE. The accuracy of SMOS was similar to that of FY-3C, and AMSR2 was the least accurate. The *r* value for FY-3C indicates that the ascent data was more accurate than descent data; for other products, the descent data was more accurate than the ascent data, and AMSR2 showed the greatest difference between ascent and descent data; Bias_r_ indicates that ascent data performed better for all products (the absolute value of Bias_r_ in ASC was smaller), and AMSR2 again showed the greatest difference; RMSE and ubRMSE were better for ascent data than descent data in SMOS and AMSR2, and for descent data in SMAP and FY-3C. We note that in SMOS the difference between ascent and descent for ubRMSE was minimal. Except for AMSR2, the satellite products were dry when compared with GLDAS data.

Quantities of data observed by each satellite over China in the period 2017–2019 were quite different due to the different satellite orbits. After exclusion using snow depth and soil temperature threshold values, the quantity of data for each product was, ranked from greatest to least, AMSR2, FY-3C, SMOS and SMAP. The quantity of data from AMSR2 was roughly equal to the total quantity of data from the other three products. The ascent data quantity was greater than the descent data quantity for both SMOS and AMSR2, and the converse was true for SMAP and FY-3C.

**Fig 1:**
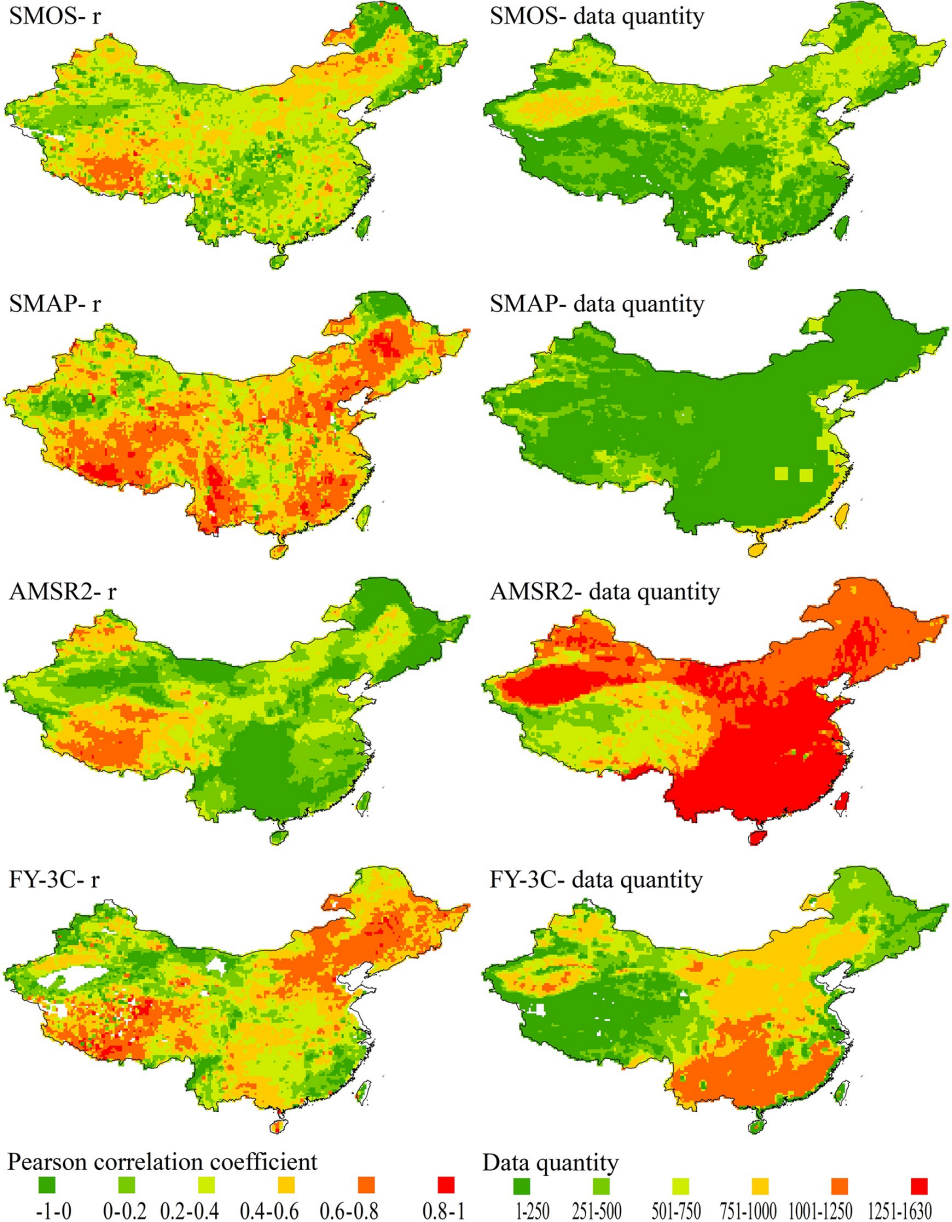
Correlation coefficients and data quantity distributions over China for passive microwave soil moisture products; grids with less than 5 data points are blank.

SMAP accurately detected surface soil moisture content in most regions of China; although it had a smaller quantity of data than the other three products, the data were evenly distributed (Fig 1). In general, the four products produced accurate soil moisture data for the Qinghai–Tibet Plateau (QTP) and parts of north and northeast China. Areas for which there was a low correlation between GLDAS and SMOS or SMAP were in parts of northeast and central China, north of QTP. Correlations between GLDAS and AMSR2 were similarly distributed, but there were more areas of low correlation. FY-3C performed better than the other three products in terms of correlation in northeast China, and areas for which it showed low correlation were in southeast coastal China and northwest and southwest China. The quantity of data observed by AMSR2 was much greater than the quantities observed by the other three products in all regions. SMOS produced more data for parts of northeast China and in the north of QTP but it was more accurate for the former area than the latter. In general, more SMOS data were observed for northern China than southern China, which is the converse of AMSR2 and FY-3C, which produced greater quantities of data for the southeast coastal areas. However, most of the regions for which there were more data did not show good correlations with GLDAS data.

Areas for which SMOS had high *r* values were in southern QTP and areas with low *r* values (negative *r*) were scattered, with most being in northeast China. Areas for which SMAP had high *r* values were in southeast and central China and areas with low *r* values were scattered, with the lowest being in central China. Areas for which AMSR2 had high *r* values were concentrated in southwest QTP and areas with low *r* values were in central China. Areas for which FY-3C had high *r* values were scattered across QTP and areas with low *r* values were scattered in western China, with the lowest *r* value being for an area near the western border. The maximum *r* values for SMOS, SMAP and FY-3C were all >0.9, and some grids in southeast region reached an *r* value of 0.99 for SMAP.

The integrity of FY-3C data was less than that of other products. There were many areas with insufficient data (<5 data points in 3 years), and there was no data for some areas in central and northern QTP. In the FY-3C product, soil water parameters for many grid squares were 0 (these areas are shown in Fig 1 as blank on the left-hand *r*-value graph and by color on the right-hand data quantity graph), such as most areas in Qaidam Basin and the sparse grid areas distributed over QTP. Some grid areas had about 1000 remote sensing soil water parameters which were all 0. All of these were excluded from the calculation of the correlation coefficient.

There were more errors in AMSR2 data than in other products. In nearly one-third of the grid areas, remote sensing soil moisture data was negatively correlated with GLDAS soil moisture data, and the negative correlation coefficient of ascent data was greater than that of descent data. There were only a few data points with negative correlations in other satellite remote sensing products.

### 3.2 Analysis of the effects of environmental factors

China is a vast country that spans many latitudes and longitudes, and environmental factors are heterogeneous in all regions of the country. In this section, we analyze in more depth the effects of topography, landcover type, and meteorological factors on the four satellite-based SM data products. The results of the analysis described in this section are significant and were obtained by multifactor analysis.

#### 3.2.1 Influence of topography

**Fig 2:**
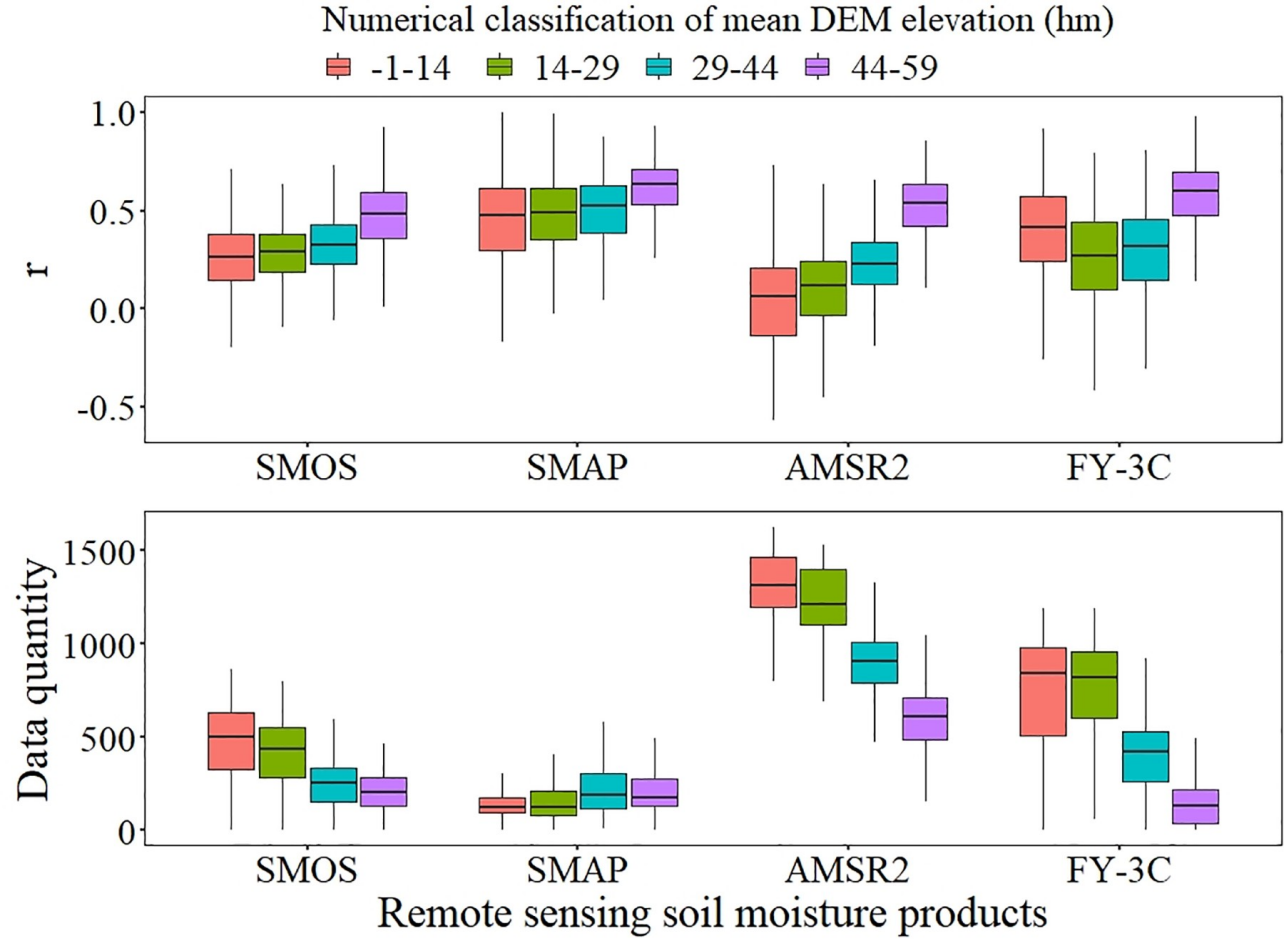
Boxplots of mean DEM elevation for *r* and data quantity.

The mean value of DEM elevation greatly affects the accuracy of microwave remote sensing SM products. The *r* values of the four products were greatest when mean DEM elevation was high (Fig 2). As mean DEM elevation increased, *r* values for SMOS, SMAP and AMSR2 increased and the *r* value of FY-3C initially decreased and then increased. These results indicated that the ability of the products to capture the trend of surface soil moisture increased for high elevations. The data quantities of SMOS, AMSR2 and FY-3C decreased as elevation increased, and the data quantity of SMAP showed a slight increasing trend.

**Fig 3:**
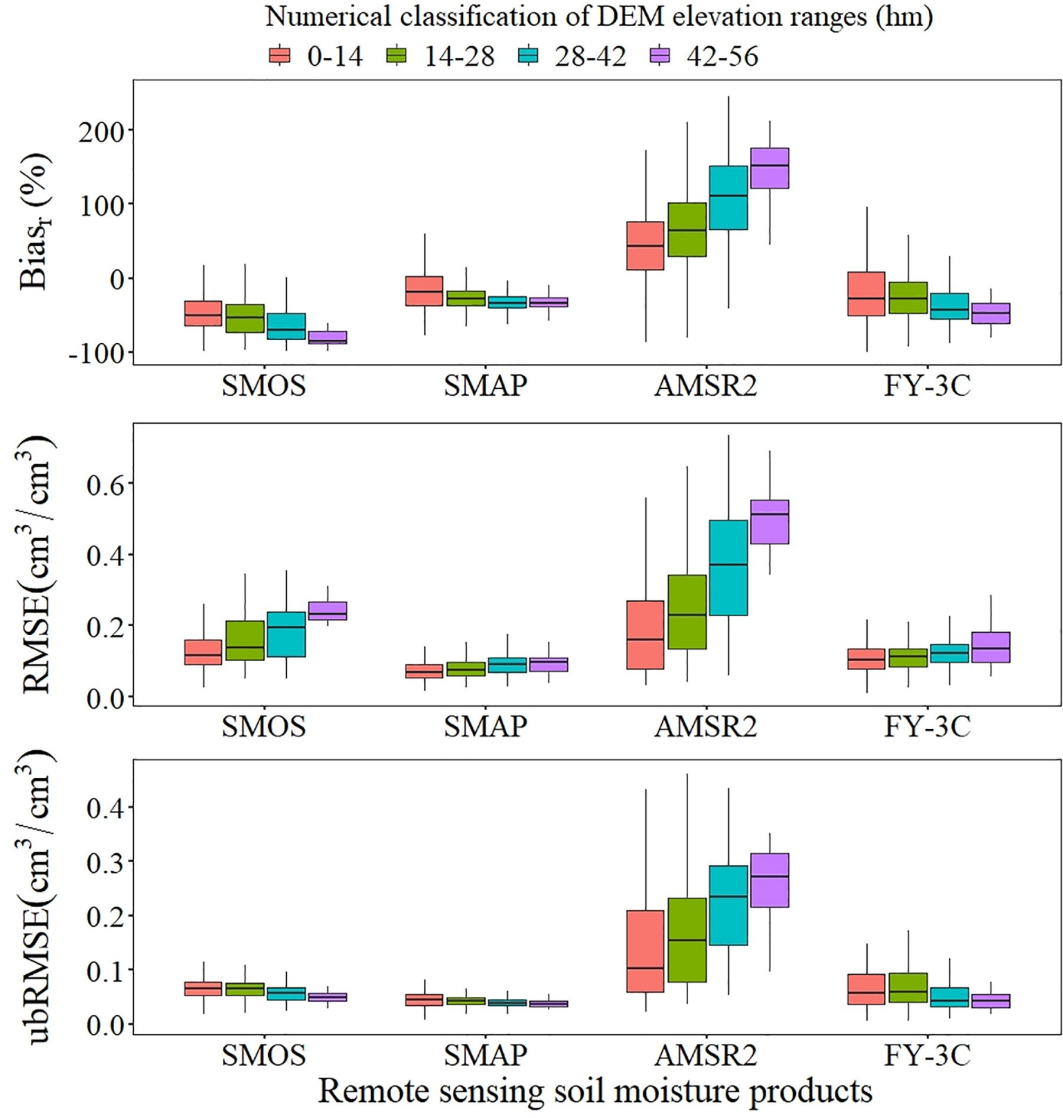
Boxplots of DEM elevation ranges for Bias_r_, RMSE and ubRMSE.

An increase in the range of DEM elevation led to an increase in bias, as indicated by Bias_r_ (in absolute value) and RMSE, in all four products. Except for AMSR2, ubRMSE had a decreasing trend (Fig 3). As the range of DEM elevation increased, the observed dryness of SMOS, SMAP and FY-3C, as indicated by Bias_r_, increased and the observed wetness of AMSR2 increased. RMSE increased for all four products; however, the trends for the increase were weak for SMAP and FY-3C. The increase in ubRMSE was significant for AMSR2, and ubRMSE showed a weak decreasing trend for SMOS, SMAP and FY-3C. The opposed trends of RMSE and ubRMSE for SMOS, SMAP and FY-3C indicated that the absolute deviation had a weak decreasing trend as the range of DEM elevation increased after removing the random error for them. The reason this phenomenon was not found in the AMSR2 product may be that there were large observation errors in this product, as indicated by Bias_r_ for high elevation exceeding 100%.

#### 3.2.2 Influence of land cover

We used the 1 km resolution land cover classification data to reassess the effects of different land cover types and variation in land cover on the performance of the four remote sensing soil moisture products. The land cover types involved in the analysis are based on post-screening data and non-water surface land covers.

**Fig 4:**
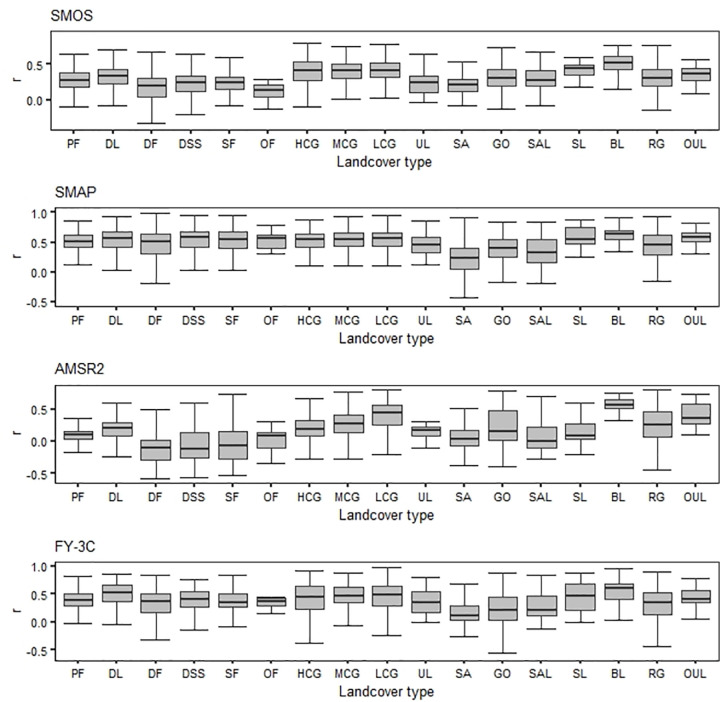
Boxplots of coefficients of correlation between SM data accuracy and land cover type for each product; abbreviations of landcover types are: PF paddy field, DL dry field, DF dense forest, DSS dwarf scrub and shrub, SF sparse forest, OF other forests, HCG high coverage grassland, MCG medium coverage grassland, LCG low coverage grassland, UL urban land, SA sand, GO Gobi, SAL saline alkali land, SL swampland, BL barren land, RG rock and gravel, OUL other unused land.

The accuracy of passively observed microwave soil moisture data is affected by differences in land cover. Different products performed differently under the same land cover (Fig 4). For cropland, forest, grassland and urban land, SMAP was more stable and significantly more accurate than either SMOS or AMSR2. For cropland, all four products were more accurate for dry field than for paddy field. For barren land (rock/sand/clay), the accuracy of SM data for the four products observed over different land covers was ordered, from low to high accuracy, sand, saline alkali land, Gobi, swampland and barren land. Accuracy varied for rock and gravel and other unused land (e.g., alpine desert and tundra). For grassland, the accuracy of AMSR2 SM data increased as grass coverage decreased, but the accuracy of the other three products did not change.

The greatest *r* value for SMOS was for rock and gravel and the least was for dense forest; the greatest *r* value for SMAP was for dense forest and the least value was for dwarf scrub and shrub; the greatest *r* value for AMSR2 was for low coverage grassland and the least value was for dense forest; both the greatest and least *r* values for FY-3C were found in low coverage grassland. We note that the best overall performance of the four surface types of products was for bare land, and the worst overall performance was for woodland (in SMOS, other forests, and in AMSR2, dwarf scrub and shrubs) and sandy land (in SMAP and FY-3C).

**Fig 5:**
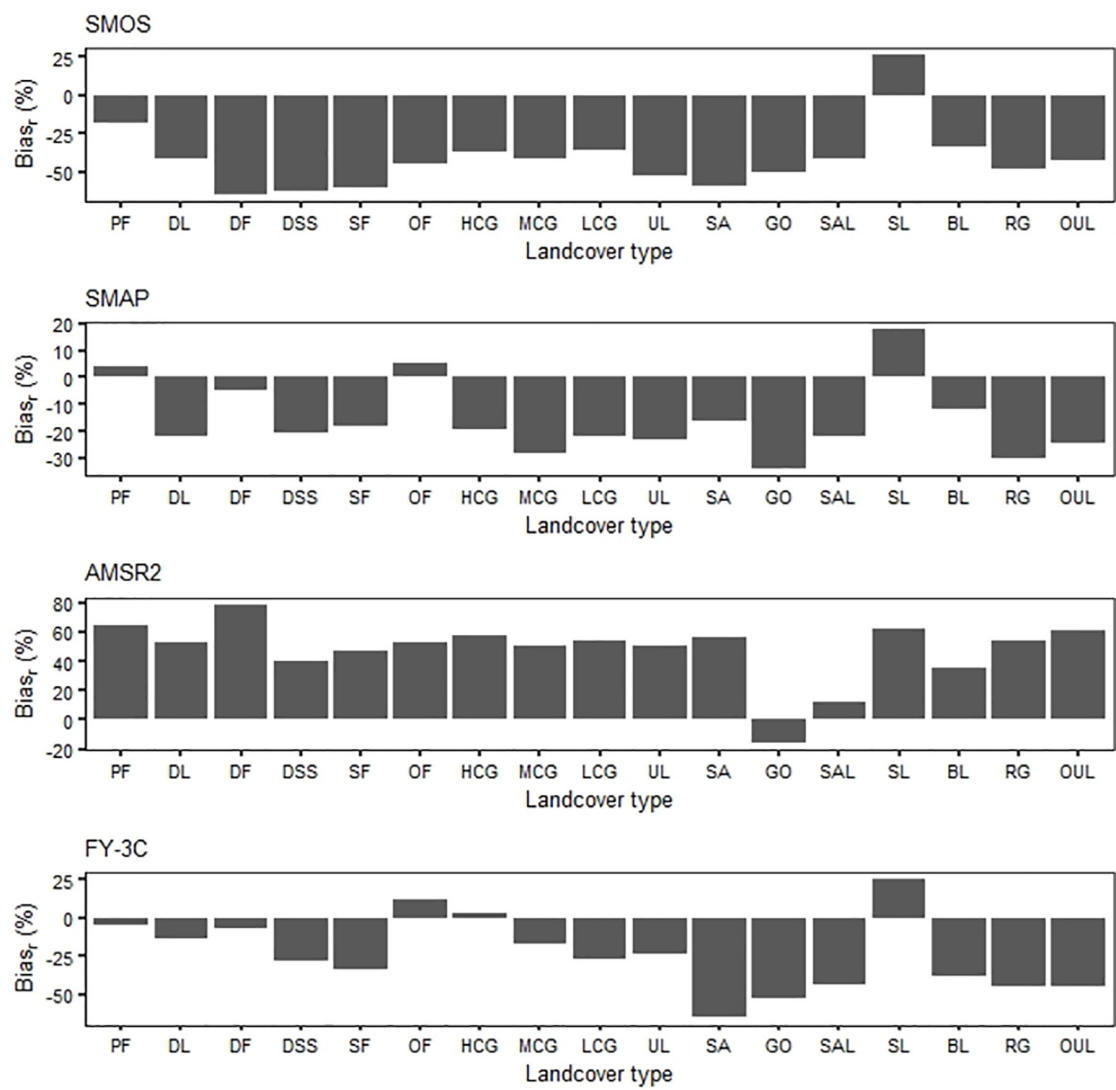
Histograms of Bias_r_ for different landcover types; the abbreviations are the same as for Fig 4.

Bias_r_ differed between products for different types of land cover. In general, the results were consistent with the overall analysis that AMSR2 was wetter and the other products drier than the GLDAS data (Fig 5). In several land cover types, Bias_r_ was consistent in direction; for example, for SM data observed on Gobi, all products were drier, and for data observed on swampland, all products were wetter. The drier trend of SMOS was greater for dense forest, dwarf scrub and shrub, sparse forest and sandy land. AMSR2 showed a much wetter trend for paddy field, dense forest and swampland. FY-3C showed a much drier trend for sand and Gobi. Bias_r_ of SMAP was overall small. Land cover types with the largest variation were medium coverage grassland, Gobi, and rock and gravel land. We note that Bias_r_ for SMAP and FY-3C in paddy field, dense forest, other forests and for FY-3C in high coverage grassland was minimal.

**Fig 6:**
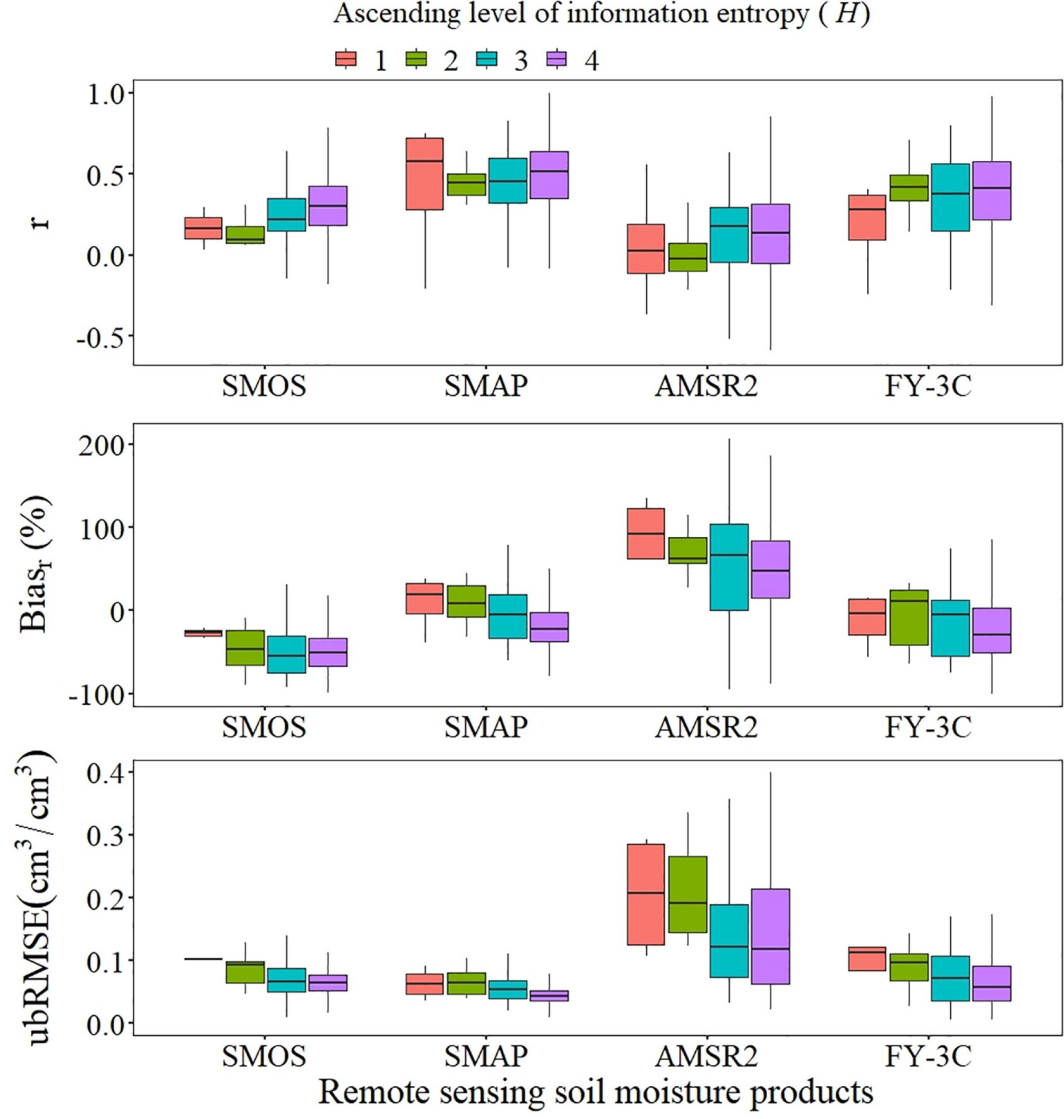
Boxplots of land cover information entropy (*H*) for r, Bias_r_ and ubRMSE.

The problem of authenticity being affected by surface heterogeneity has become a major research issue because of the low resolution of passive microwave remote sensing data [31]. Information entropy indicates the extent of uncertainty or randomness in a variable, and it was used in our analysis of the effects of surface heterogeneity on SM data accuracy for the four products. The results show that overall the observation data of microwave remote sensing products is more accurate when land surface information entropy increases; that is, increased heterogeneity of land surface type indicates greater observation accuracy but increased fluctuation in error coefficients (Fig 6). AMSR2 was the most typical. As land cover heterogeneity increased, the *r* value of AMSR2 increased and Bias_r_ and ubRMSE decreased. As information entropy increased, the *r* value of SMAP varied slightly, Bias_r_ indicated a change from wetter to drier, and ubRMSE tended to decrease. For both SMOS and FY-3C, observed SM increased as information entropy increased; that is, *r* increased and ubRMSE decreased, but the dryness indicated by Bias_r_ increased.

#### 3.2.3 Influence of meteorology

Meteorological parameters are environmental factors that affect remote sensing SM observation [32]. We used T_s_ for the 0–10 cm soil layer and daily precipitation data to determine the effects of meteorological factors on SM data for each product. The meteorological parameters used in the analysis were calculated using all meteorological data for each grid pixel from 2017 to 2019 and were then used as the reference values for meteorological conditions.

**Fig 7:**
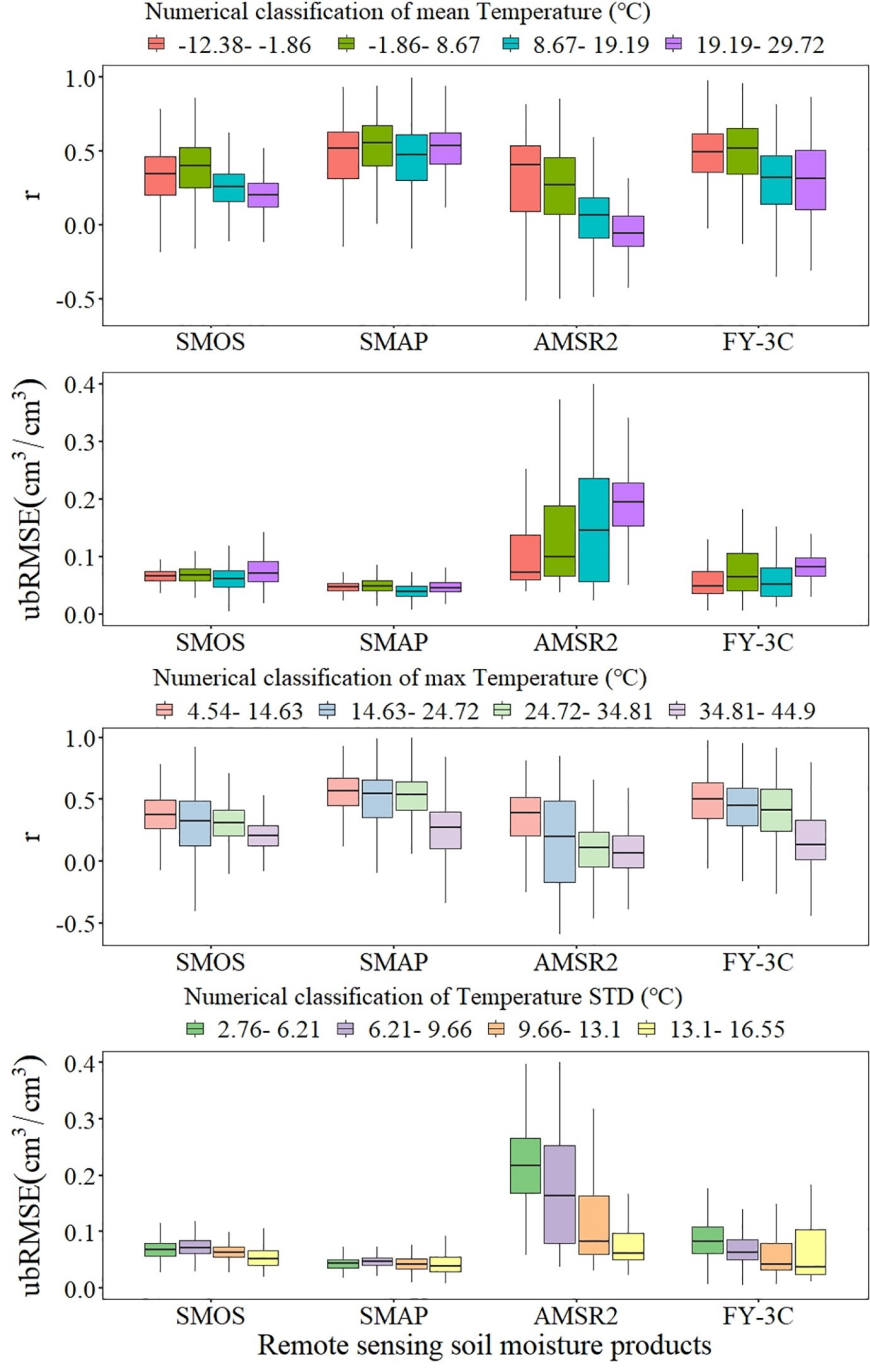
Boxplots of temperature condition for *r* and ubRMSE.

The *r* values of SM of SMOS, AMSR2 and FY-3C all decreased as mean temperature increased; the accuracy of SMAP was unchanged. However, the *r* values of all products decreased as maximum temperature increased, and ubRMSE for all products except AMSR2 did not change significantly as the temperature indicators changed (Fig 7). When the mean temperature of the grid was low, the *r* value increased for all products. When the mean temperature of the grid was high (19.19–29.72°C), only SMAP gave a reliable value, but when the maximum temperature of the grid reached 34.81–44.9°C, the *r* value reached a minimum for all products. The absolute deviation (ubRMSE) of other products did not fluctuate significantly with temperature factors when compared with AMSR2. The ubRMSE of AMSR2 increased significantly as mean temperature increased but decreased significantly as the standard deviation of temperature increased, which indicated that the observation error of AMSR2 increased at higher temperatures but that the observation accuracy of AMSR2 increased as variation in temperature increased.

**Fig 8:**
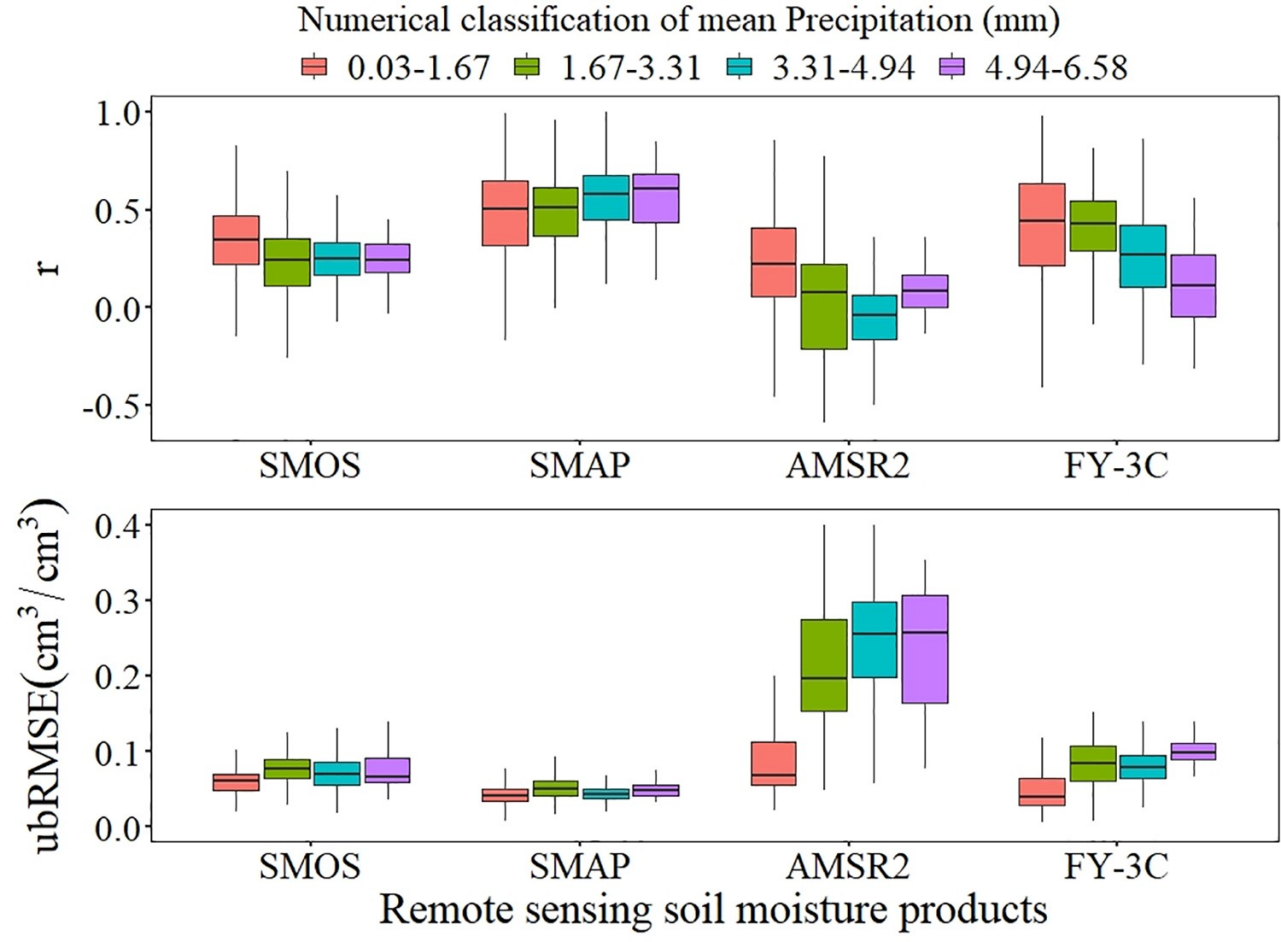
Boxplots of precipitation condition for *r* and ubRMSE.

The *r* values of SMOS and SMAP were only slightly affected by precipitation. The *r* value of AMSR2 initially decreased and then increased, and ubRMSE gradually increased, as precipitation increased. The observation accuracy of FY-3C decreased as precipitation increased (Fig 8).

Areas with large coefficients of determination were generally regions showing low mean temperatures (−1.86–8.67°C), maximum temperatures ≤34.81°C, and little precipitation (0.03–1.67 mm).

### 3.3 Triple collocation analysis

Soil surface moisture is a most challenging land surface parameter to observe accurately in remote sensing quantitative retrieval [33]. TCA can overcome the difficulty of obtaining standard soil moisture data at a regional scale with high temporal and spatial resolution in evaluation studies. The preceding analysis showed that the performance of the SMAP SM product in China was optimal among the four remote sensing products. We therefore engaged in TCA of combinations SMOS-GLDAS-SMAP, AMSR2-GLDAS-SMAP and FY-3C-GLDAS-SMAP. For the grids in which all matched products were positively correlated, the performance was analyzed by error component.

**Fig 9:**
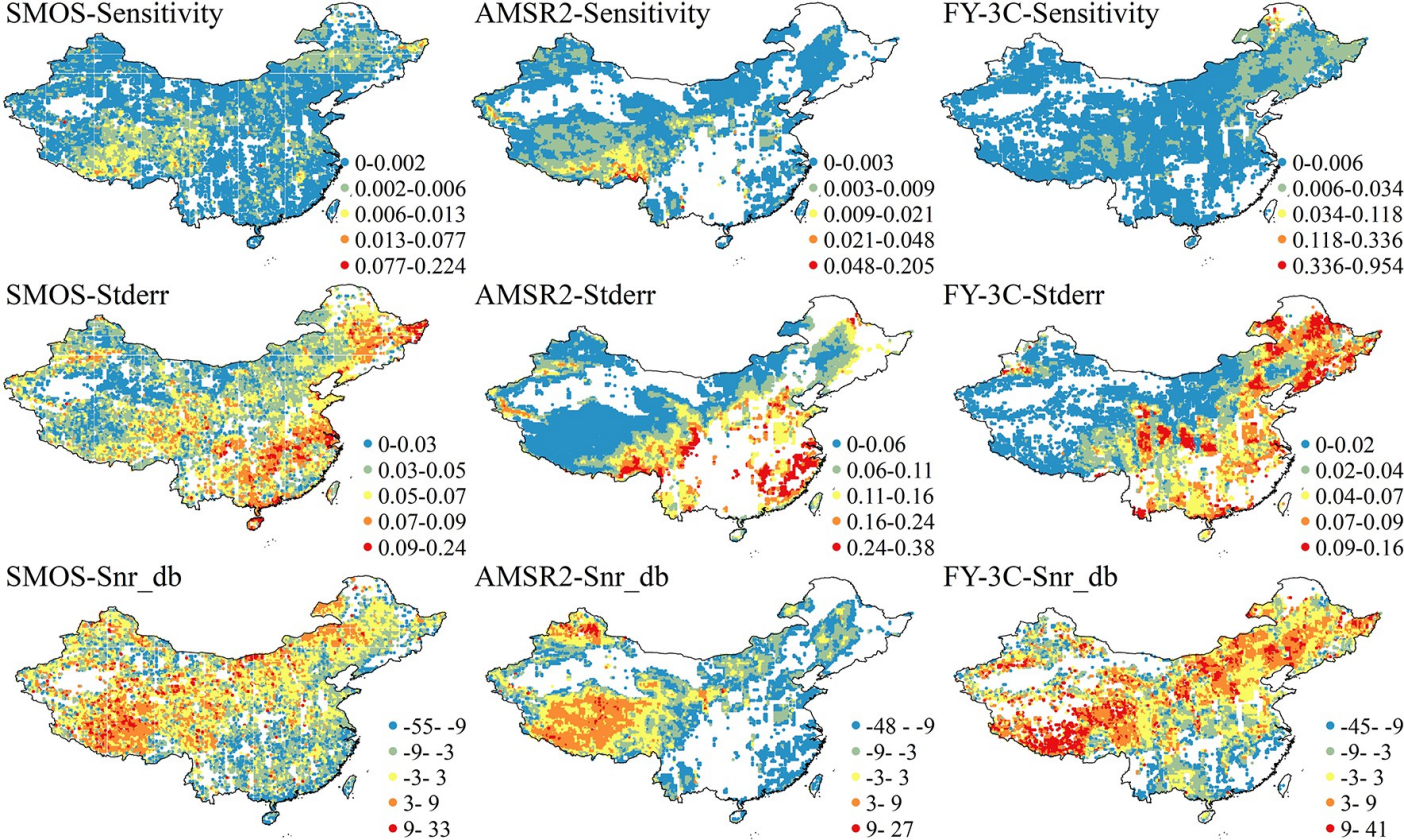
Distribution of parameters across China for SMOS, AMSR2 and FY-3C soil moisture products given by triple collocation analysis; results are only shown for grids which all three datasets were positively correlated.

The results show that SMOS and AMSR2 in most areas of the Tibetan Plateau, FY-3C in some areas of the Tibetan Plateau, and SMOS and FY-3C in some areas of northeast China showed high Sensitivity. Overall, Sensitivity was high for FY-3C, and the highest value appeared in northeast China (Fig 9). SMOS, AMSR2 and FY-3C all showed high values of Stderr in parts of southeast and northeast China and mostly low values in western and northern China. AMSR2 had a high error level. The signals of surface soil moisture detected by remote sensing contain noise due to many factors. Noise in SMOS and FY-3C was weak in most areas of western, northern and northeastern China and noise in AMSR2 was weak in the Qinghai–Tibet Plateau and the northwest border region. However, noise had a great effect on soil moisture in the southeastern region, especially coastal areas. Noise from the AMSR2 signal was relatively high in the three products. The reliability of the detection results for the three products was low in the following areas, as shown in the blank areas for SMOS in the northwest of the Qinghai–Tibet Plateau, AMSR2 in the west, central and south, and northeast of China, and FY-3C in the west, southeast and northeast parts of China in the TCA results.

## 4. Discussion

### 4.1 Causes of errors in authenticity testing

The validation and assessment of passive microwave soil moisture products are important and challenging tasks. The differences in sensor performance, sensing depths, spatial resolutions, retrieval algorithms and inputs, amongst others, contribute to the difficulties of comparing soil moisture products [2]. In validating four mainstream passive microwave remote sensing soil moisture products, we found that the factors that generate uncertainty were: (1) differences in spatial resolution between different products [34]; (2) differences in observation frequency and differences in detection technology between satellite-borne sensors [35]; (3) differences in input data and soil moisture data retrieval algorithms between products [23]; and (4) the representation of GLDAS soil water parameters [36].

Difference in spatial scales is one of the key issues in determining the authenticity of remote sensing data products [37]. An accurate assessment of a product requires the use of realistic data processing methods. We used the GLDAS dataset to avoid the problems with the representation of ground observations that are found in conventional verification methods and we matched data according to the transit times of satellites to avoid errors due to differences in times of observation [28]. The use of TCA reduced the effects of differences in scale on the evaluation [38]. The spatial scale mismatch between different soil moisture products was particularly acute in the lower spatial resolution of SMAP (36 km), which we resampled using inverse distance weighting [39]. Comparison with other products showed that the change in scale did not have a significant impact on product performance.

The characteristics of each satellite-borne sensor, such as its detection frequency, must be carefully examined in a comprehensive comparison of each product. It is generally accepted that L-band is best for soil moisture detection because it is more sensitive to SM than other bands and more easily penetrates vegetation [37]. It is also less sensitive to atmospheric changes, such as heavy rain, than the high-frequency and short-wavelength C- and X-bands. L-band penetration depth (5 cm) covers approximately half of the modeled depth of GLDAS (0–10 cm), but it is greater than X-band penetration depth, and as SM increases the microwave penetration depth decreases [40]. These characteristics suggest that L-band products (SMOS and SMAP) should perform better than X-band products (AMSR2 and FY-3C), but performance differences were by no means entirely due to differences in sensor frequencies.

Different observational techniques also result in differences in observation. For example, although both SMAP and SMOS use L-band to generate soil moisture data, the SMAP instrument is a real aperture radiometer whereas SMOS uses a synthetic aperture radiometer; thus SMOS observation produces more internal noise [38]. The relationship between TB and incidence angles is used in the data retrieval algorithm of SMOS to decrease internal noise, but data retrieval is also disrupted by RFI, the extent of which is unknown before launch. In contrast, SMAP provides observation data for a particular location at a fixed incidence angle, which likely contributes to the decreased noise in the retrieved data, as confirmed in our analysis.

It is important to determine how different factors affect SM products in order to improve the quality of retrieved data. Knowing how each factor influences error can help to explain its contribution to noise. If a factor is strongly correlated with error(s) in the input factors of the retrieval algorithm, the model related to it should be improved; if the factor is not related to an input factor of the retrieval algorithm, it may improve the accuracy of the product if the factor is incorporated in the retrieval algorithm [39]. For example, the meteorological element T_s_ is an important input parameter of the SM retrieval algorithm, and precipitation directly changes soil moisture [40]. The accuracy of realtime determination of a satellite soil moisture product will be improved if meteorological factors are better incorporated into the retrieval process [32]. We found that the accuracy of SM data retrieved by AMSR2 clearly showed the greatest decreasing trend for mean temperature (Fig 7). The parameter T_s_ was calculated in the AMSR2 retrieval algorithm based on the empirical relationship between T_s_ and the vertical linear (V) polarization of TB [41]; improving the calculation of T_s_ would increase SM data accuracy for AMSR2.

The authenticity of SM data provided by the four products was determined through data analysis and comparison to reference data. The analysis provided quantitative information about data quality by estimating systematic and random errors. In this process, the reference data are presumed to closely represent truth (i.e., they are considered to be accurate) [42]. However, due to the coarse resolution of microwave remote sensing products, it is almost impossible to obtain high quality reference data covering a large variety of climatic, topographic, and land cover conditions [43]. The use of GLDAS data avoids the systematic errors associated with the use of a single point observation as representative of an entire area, and GLDAS data can therefore be used as reference data for remote sensing soil moisture products over wide areas. Although GLDAS parameters do not present the true level of surface soil moisture, they are still considered to be reference data for remote sensing soil moisture products in relevant studies [43–45]. Moreover, TCA, as demonstrated in this study, can overcome the problem of lack of accurate reference data and thus provide more rigor to the study.

### 4.2 Selection of optimal product and impact factors for China

There was both consistency and difference in performance of the different products in retrieving data for China. The high correlations and low error levels in the evaluation parameters found between each product and GLDAS data and the ability of each product to accurately measure surface soil moisture as indicated by the TCA error components together indicate that satellite-borne passive microwave detection can accurately observe surface soil moisture.

The results shown in Fig 1 and Table 2 indicate that the different products perform differently in observation across China, and that their accuracy and the quantity of data they provide differs according to orbit and region. Analysis of a variety of error parameters and other factors, including topography, land cover classification, and meteorological factors, showed that they all affect SM data accuracy, but that they differ in the extent of their effects.

The deviation from observation values represented by Bias_r_ is affected by many factors besides representation errors caused by scale mismatch, errors inherent in the retrieval algorithm, and error amplification [35, 46–48]. These other factors include different sensing depths (and thus differences in the quantity of observed data), the use of auxiliary variables from models (e.g., soil temperature), uncertainty in the reference data, the scaling and conversion of units (mainly for GLDAS), and spatial heterogeneity. In addition, error determination should consider the own value. If the soil moisture parameter in a pixel has a high value, a relatively high observation error can be tolerated. However, if the soil moisture parameter has a low value, even a very low observation error can have a great effect on the estimation of the error. We used the relative value of the observation bias as an error evaluation parameter, and this value can be used as an indicator of the dryness or wetness (i.e., the degree of SM) of the observation results and thus the degree of deviation from truth. All products except AMSR2 erred on the side of dryness for China, and the bias degree from high to low was ordered as AMSR2, SMOS, FY-3C and SMAP (Table 2). All products were drier in Gobi and wetter in swampland, and the microwave detection of soil moisture deviates from truth particularly in dense forest, sand and Gobi (Fig 5).

The SM data provided by SMAP using 1.4 GHz band detection were overall more accurate for China than the data from other products. This finding is consistent with other studies [49, 50] and it is likely due to the greater capability of the sensor and the better RFI filtering algorithm [17]. SMAP SM data were slightly less well correlated for sand and Gobi (Figs 4 and 5), but their accuracy was little affected by environmental factors, as Ma et al. [51] also found.

The soil moisture product of FY-3C, independently developed in China, generally performed in the middle of the four products, but its overall quality was reduced by poor data quality in some regions and by some grids showing negative correlations. Its poor observation accuracy in Gobi and sand is likely due to the large systematic error in observation. The accuracy of FY-3C was easily affected by precipitation conditions. Its performance in capturing SM in northeast China was relatively good, but it also showed high variability in observation.

Of the many factors considered, vegetation cover and surface roughness greatly affect the accuracy of remote sensing soil moisture observation [23, 52]. The land use classification that provided the most accurate data was barren land (Figs 4 and 5), which is consistent with other research [53]. The accuracy of SM data showed the same basic change trend for all four products with changes in secondary land cover types of cropland and barren land (rock/sand/clay). The highest correlations between AMSR2 and FY-3C and the reference data were found for grassland, and the lowest correlations of SMOS, SMAP and AMSR2 were found for forest. The landcover types that produced the worst overall accuracy were forest and sand. Other studies have confirmed that the limited range of soil moisture in deserts and forested areas contributes to higher errors for these areas [50].

The distribution of high values of Snr_db for the three products almost matches the distribution of the *r* value. Areas of high Sensitivity for SMOS and AMSR2 overlapped with areas of high *r* values; areas of high Sensitivity for FY-3C overlapped with areas of high *r* values in northeast China and partially overlapped with them in the Qinghai–Tibet Plateau, although the areas of relatively high *r* values in southern China were not matched in the Sensitivity distribution. The high Sensitivity areas of SMOS and AMSR2 were similar in distribution to areas of high values of Snr_db but opposite to the distributions of Stderr. We note that FY-3C shows areas of high values of Sensitivity, Stderr and Snr_db in northeast China.

When we combined the above results to analyze product performance, we found that the best performance in terms of SM data accuracy from passive microwave remote sensing was obtained when the terrain was higher in elevation and the surface was uniform, with soil underlying the surface, a lower mean temperature and less precipitation. The best performance was produced when the upper vegetation layer was simple and the moisture content was low. The performance of all products was good for the QTP region because the region has characteristics in common with regions that show high correlations for multiple factors.

## 5. Conclusions

In this study, the assimilated GLDAS dataset was used as baseline data for an evaluation and comparison across China of four mainstream passive microwave SM data products (SMOS, SMAP, AMSR2 and FY-3C). The comparison embraced a range of different topographies, land covers and meteorological conditions. The indicators used for assessment of the products included the Pearson correlation coefficient (*r*), relative bias (Bias_r_), root mean square error (RMSE), unbiased root mean square error (ubRMSE); Sensitivity, standard error (Stderr), and signal-to-noise ratio in decibels (Snr_db) of triple collocation analysis provided metrics. All data was processed using established methods, and the results we obtained were realistic and credible. They, therefore, have value as reference data for other studies. Several conclusions were drawn from the research, and the most important results are presented in the following paragraphs.

SMAP soil moisture products produced more accurate data for China than other products and produced accurate SM data for most regions of China. Observational accuracy varied less than for other products due to environmental factors. SMAP performed only slightly worse for land cover types sand and Gobi and maximum temperatures in the range 34.81–44.9°C.

Passive microwave soil moisture detection technology performs better in terms of data accuracy when the terrain is at a higher elevation and the surface is uniform, the underlying surface is soil, the mean temperature is lower and there is less precipitation. These criteria are satisfied for the Qinghai–Tibet Plateau, but across most of China, most soil moisture parameters are underestimated.

Environmental factors can affect the performance of microwave remote sensing soil moisture products, especially AMSR2. Subsequent improvement of its retrieval algorithm should include optimization by combining topography, land cover, and meteorological factors, and more localized improvements can be made for western, southeast and northeast China.

FY-3C is China’s second-generation polar-orbiting meteorological satellite. It performed well in our evaluation, with overall accuracy similar to that of SMOS but less than that of SMAP. It provides accurate SM data for the Qinghai–Tibet Plateau, central China, and particularly for northeast China, where its SM data is more accurate than other products; however, the SM data it provides are of low integrity and quality for some regions.
